# Iodic Acid a Test for the Vegeto-Alkalies

**Published:** 1831

**Authors:** 


6. *Iodic Acid a test of the Vegeto Alkalies.*—Mr. Serullas has shown, that the smallest quantity of the new vegetable alkalies, may be detected by iodic acid, or by a solution of perchloride of iodine, because of the iodic acid there present, or ready to be formed. The sensibility is so great that the acid may be regarded as one of the most delicate tests in use. The hundredth part of a grain of quinia or cinchonia, dissolved in several thousand times its weight of alcohol, may in this way be precipitated, and the precipitate quickly collected. All the vegeto-alkalies are not equally well tested in this way, but the least sensible is discovered, if one-fifth of a grain is present.—*An. de Chim. Vol. XIV.*

